# Imiquimod Induces Apoptosis of Squamous Cell Carcinoma (SCC) Cells via Regulation of A20

**DOI:** 10.1371/journal.pone.0095337

**Published:** 2014-04-17

**Authors:** Kyung-Cheol Sohn, Zheng Jun Li, Dae-Kyoung Choi, Tiejun Zhang, Jae Woo Lim, In-Kyu Chang, Gang Min Hur, Myung Im, Young Lee, Young-Joon Seo, Jeung-Hoon Lee, Chang Deok Kim

**Affiliations:** 1 Department of Dermatology and Research Institute for Medical Sciences, School of Medicine, Chungnam National University, Daejeon, Korea; 2 Department of Pharmacology, School of Medicine, Chungnam National University, Daejeon, Korea; Juntendo University School of Medicine, Japan

## Abstract

Imiquimod, a nucleoside analogue of the imidazoquinoline family, is being used to treat various cutaneous cancers including squamous cell carcinoma (SCC). Imiquimod activates anti-tumor immunity via Toll-like receptor 7 (TLR7) in macrophage and other immune cells. Imiquimod can also affect tumor cells directly, regardless of its impact on immune system. In this study, we demonstrated that imiquimod induced apoptosis of SCC cells (SCC12) and A20 was involved in this process. When A20 was overexpressed, imiquimod-induced apoptosis was markedly inhibited. Conversely, knockdown of A20 potentiated imiquimod-induced apoptosis. Interestingly, A20 counteracted activation of c-Jun N-terminal kinase (JNK), suggesting that A20-regulated JNK activity was possible mechanism underlying imiquimod-induced apoptosis of SCC12 cells. Finally, imiquimod-induced apoptosis of SCC12 cells was taken place in a TLR7-independent manner. Our data provide new insight into the mechanism underlying imiquimod effect in cutaneous cancer treatment.

## Introduction

Squamous cell carcinoma (SCC) is one of epithelial cancers, which is originated from the upper layers of skin epidermis. The incidence of SCC is relatively high, ranking as the second most frequent type among the non-melanoma skin cancers [1]. Ultraviolet (UV) radiation is the best-known cause of SCC, which primarily affects DNA thereby inducing mutations of many susceptible genes including p53 [2]. Intracellular signal regulators such as epidermal growth factor receptor (EGFR), Src-family tyrosine kinase Fyn, and nuclear factor κ-light-chain-enhancer of activated B cells (NF-κB) are also implicated in the development of SCC [3]–[6]. For example, blockade of NF-κB promotes SCC in both murine and human skins, highlighting its pivotal role in maintenance of skin homeostasis [5], [6].

Imiquimod (R-837) is an immune response modifier, activating macrophage and other cells via Toll-like receptor 7 (TLR7). Imiquimod provokes Th1 cell-mediated immune response via inducing the secretion of proinflammatory cytokines such as interferon-α (IFN-α), tumor necrosis factor-α (TNF-α), and interleukin-12 (IL-12) [7], [8]. Currently, imiquimod as a 5% cream is used to treat several skin diseases, including malignant melanoma, basal cell carcinoma (BCC), and SCC [9]–[11]. With respect to SCC treatment, it has been demonstrated that imiquimod stimulates tumor destruction by recruiting cutaneous effector T cells from blood and by inhibiting tonic anti-inflammatory signals within the tumor [12]. Other evidence shows that topical imiquimod treatment attenuates the de novo growth of UV-induced SCC through activation of Th17/Th1 cells and cytotoxic T lymphocytes [13]. In addition to its immune-modulatory effect, imiquimod has been shown to activate keratinocytes by binding to adenosine receptors in keratinocytes, independently of TLR7 [14]. Thus, we hypothesize that imiquimod has direct effect on SCC cells, regardless of its impact on immune system.

As notified, NF-κB is the important key player in the control of keratinocyte growth and carcinogenesis. The activity of NF-κB is strictly controlled by sophisticated network of negative and positive regulators. We found that A20, one important negative regulator for NF-κB, was highly increased in SCC cells. Since imiquimod affects NF-κB pathway in a TLR-dependent and/or -independent manner in other systems, we investigate whether the effect of imiquimod is related with A20 in SCC cells. Our data provide evidence that imiquimod induces apoptosis of SCC cells via regulation of A20.

## Materials and Methods

### Ethics Statement

This study was approved by the Institutional Review Board of Chungnam National University Hospital. All human skin samples were obtained under the written informed consent of donors.

### Reagents and Antibodies

Imiquimod was purchased from Santa Cruz Biotechnologies (Santa Cruz, CA). The following primary antibodies were used in this study: A20 (Calbiochem, La Jolla, CA), PARP (BD Biosciences, San Jose, CA), caspase-3, ERK, phospho-ERK, JNK, phospho-JNK, p38 MAPK, phospho-p38 MAPK (Cell Signaling Technology, Beverly, MA), TLR7 (Enzo Life Science, Farmingdale, NY), GFP (Santa Cruz Biotechnologies), actin (Sigma-Aldrich, St. Louis, MO).

### Immunohistochemistry

Paraffin sections were dewaxed, rehydrated, then washed three times with phosphate-buffered saline (PBS). After treatment with proteinase K (1 mg/ml) for 5 min at 37°C, sections were treated with H_2_O_2_ for 10 min at room temperature, blocked in 0.1% Tween-20, 1% bovine serum albumin (BSA) in PBS for 30 min, and followed by reaction with appropriate primary antibodies. Sections were incubated sequentially with peroxidase-conjugated secondary antibodies and visualized with Chemmate envision detection kit (Dako, Carpinteria, CA).

### Cell Culture

SV40-transformed human epidermal keratinocytes (SV-HEK), melanocytes and fibroblasts were cultured according to the methods previously reported [15]. SCC12 and SCC13 cells were maintained in Dulbecco’s modified Eagle’s medium (DMEM) supplemented with 10% fetal bovine serum (FBS) (Life Technologies Corporation, Grand Island, NY). For viability test, SCC12 cells were seeded in 6 well plate at a density of 2×10^5^, treated with imiquimod for 24 h, then MTT assay was performed.

### Western Blotting

Cells were lysed in Proprep solution (Intron, Daejeon, Korea). Total protein was measured using a BCA Protein Assay Reagent (Pierce Biotechnology, Rockford, IL). Samples were run on SDS-polyacrylamide gels, transferred onto nitrocellulose membranes and incubated with appropriate antibodies. Blots were then incubated with peroxidase-conjugated secondary antibodies, visualized by enhanced chemiluminescence (Intron).

### Detection of Apoptosis

Apoptosis was detected using FITC annexin V apoptosis detection kit (BD Biosciences). After treatment of imiquimod, cells were washed twice with cold PBS and stained with FITC annexin V and propidium iodide (PI). Cells were then analyzed by flow cytometry.

### Adenovirus Creation

Total RNA was isolated from human embryonic kidney cells 293A and used for cloning of A20 cDNA fragment. The primer set for A20 is as follows: forward 5′-AGATCTATGGCTGAACAAGTCCTTCC-3′, and reverse 5′-CTCGAGTTAGCCATACATCTGCTTG-3′. The amplified full-length cDNA for A20 was subcloned into the pENT/CMV-GFP vector that had attL sites for site specific recombination with a Gateway destination vector. Replication-incompetent adenoviruses were created using the Virapower adenovirus expression system (Invitrogen). The adenovirus was purified with cesium chloride [16].

### Knockdown of Gene Expression

For knockdown of A20 expression, we used lentivirus expressing short hairpin RNA (shRNA). The shRNA plamid DNA stocks (SHCLNG-NM_006290) were purchased from Sigma-Aldrich (St Louis, MO), and recombinant lentivirus was produced as previously reported [17]. The shRNA sequences were as follows: #1, 5′-CCGGCACTGGAAGAAATACACATATCTCGAGATATGTGTATTTCTTCCAGTGTTTTTG-3′; #2, 5′-GTACCGGAGTTGGATGAAGCTAACTTACCTCGAGGTAAGTTAGCTTCATCCAACTTTTTTTG-3′. SCC12 cells were transduced with lentivirus, then stable cells expressing shRNA-A20 were selected by puromycin treatment. In parallel, stable cells expressing shRNA-scrambled (shRNA-Scr) was also established as a negative control.

For microRNA (miR) specific for TLR7, target sequences were designed using Invitrogen’s RNAi Designer. The double-stranded DNA oligonucleotides were synthesized and cloned into the parental vector pcDNA6.2-GW/EmGFP-miR (Invitrogen, Carlsbad, CA). The expression cassette for miR was moved into pENT/CMV vector, and then adenovirus was made as previously reported [18]. The miR sequence was as follows: 5′-TGCTGTGAAATCGATCTCTACCAGATGTTTTGGCCACTGACTGACATCTGGTAGATCGATTTCA-3′.

## Results

### Expression of A20 in SCC

**Figure 1:**
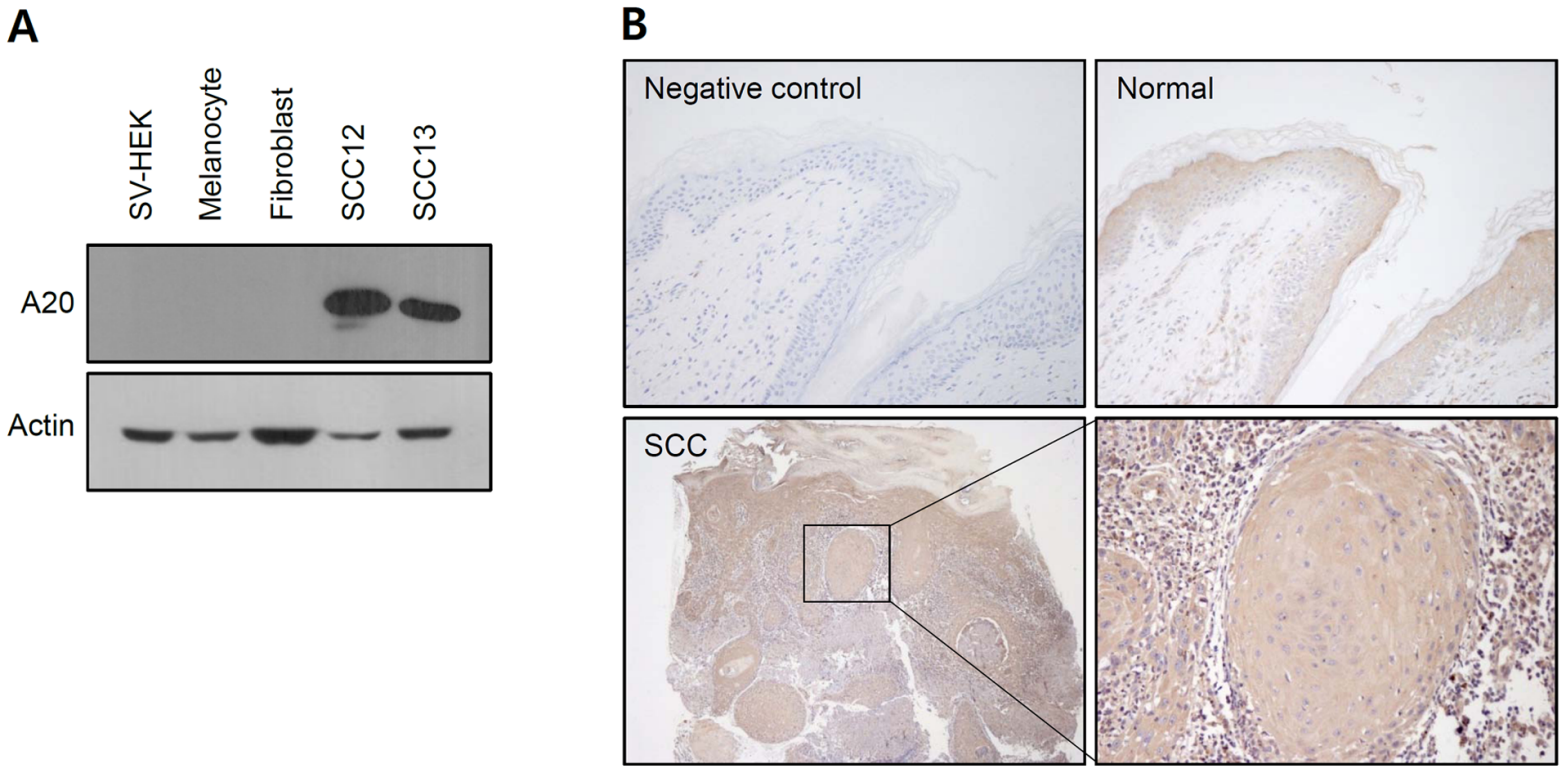
Expression of A20 in skin cells. (A) Expression of A20 was detected using Western blot analysis. High expression of A20 is observed in squamous carcinoma cell line SCC12 and SCC13. (B) Expression of A20 was detected in skin tissues by immunohistochemistry. In normal skin, A20 expression is increased in upper layers of epidermis. In SCC, high expression of A20 is detected in cancer lesions. In negative control, primary antibody was omitted.

NF-κB is a regulator for antiapoptotic and proinflammatory responses, and recognized as an important player in SCC [19], [20]. In a preliminary experiment, we found that large number of NF-κB target genes was up-regulated in SCC cell line SCC12, as compared with normal human epidermal keratinocytes (NHEK) (Figure S1). We focused on one interesting target molecule A20 (also known as TNFAIP3), which is a feedback inhibitor for NF-κB activation [21], [22]. We first compared the A20 expression in cultured skin cells, and found that protein level for A20 was markedly increased in SCC cell lines, such as SCC12 and SCC13 cells (Figure 1A). In immunohistochemistry analysis, A20 expression was not detected in basal layer of normal epidermis, while increased expression of A20 was observed in upper layers of normal epidermis. In SCC lesions, high level A20 was detected by immunohistochemistry (Figure 1B). We observed moderate to high expression of A20 in more than 70% patient samples by tissue array analysis (Figure S2).

### Imiquimod Induces Apoptosis of SCC12 Cells by Suppressing A20 and Activating JNK

**Figure 2:**
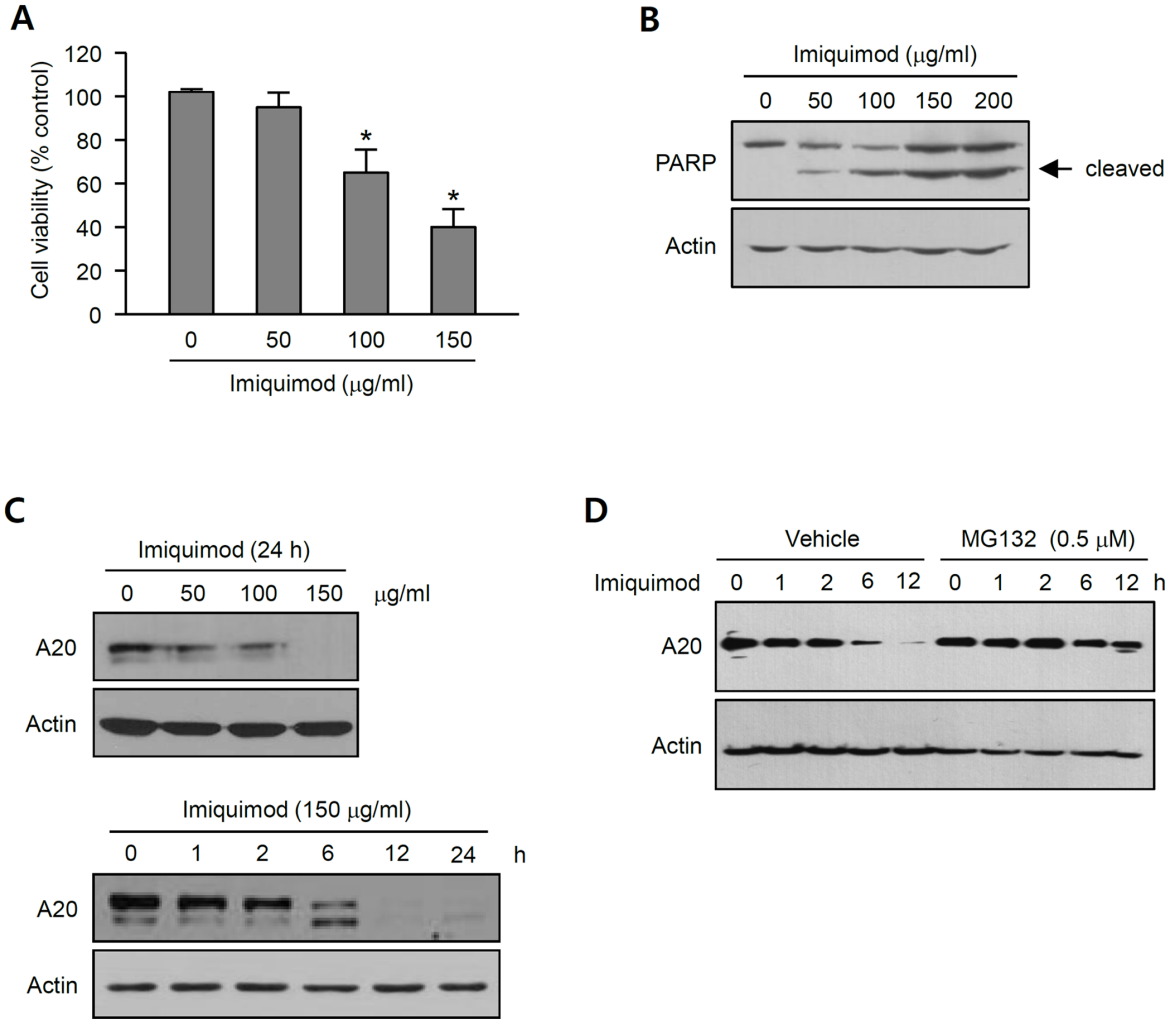
Imiquimod-induced apoptosis of SCC12 cells. (A) Cells were treated with imiquimod at the indicated concentrations for 24 h. Cell viability was determined by MTT assay. Data are expressed as percentage of control (0 µg/ml imiquimod). The mean values ± SD are averages of triplicate measurements. (B) To determine whether imiquimod induces apoptosis of SCC12 cells, cleavage of PARP, a prominent feature of the apoptotic execution phase, was checked by Western blot. Anti-actin antibody was used as a loading control. Imiquimod induces cleavage of PARP in a dose-dependent manner. (C) Cells were treated with imiquimod at the indicated concentrations and/or for the indicated time points. Expression of A20 was detected by Western blot. Imiquimod induces down-regulation of A20 in a dose- and time-dependent manner. (D) Cells were pretreated with MG132 then treated with imiquimod (150 µg/ml) for the indicated time points. MG132 blocks imiquimod-driven A20 down-regulation.

Imiquimod is being successfully used for treatment of SCC. As notified, imiquimod can affect keratinocytes independently of immune system, we investigated direct effect of imiquimod on SCC cells. When SCC12 cells were treated with imiquimod, cell death was occurred in a dose-dependent manner (Figure 2A). Western blot showed that imiquimod treatment resulted in cleavage of poly (ADP-ribose) polymerase (PARP), confirming the imiquimod-induced apoptosis of SCC12 cells (Figure 2B). In addition, imiquimod treatment led to cleavage of caspase-9, but not caspase-8, suggesting that imiquimod induces activation of intrinsic apoptotic pathway (Figure S3). Interestingly, imiquimod treatment led to marked down-regulation of A20 in a dose- and time-dependent manner (Figure 2C). This imiquimod-driven A20 down-regulation, however, was markedly prevented by pretreatment with proteasome inhibitor MG132, indicating that imiquimod induces the degradation of A20 in a proteasome-dependent fashion (Figure 2D). Together, these data suggest that imiquimod-induced apoptosis of SCC12 cells may occur via the regulation of A20.

**Figure 3:**
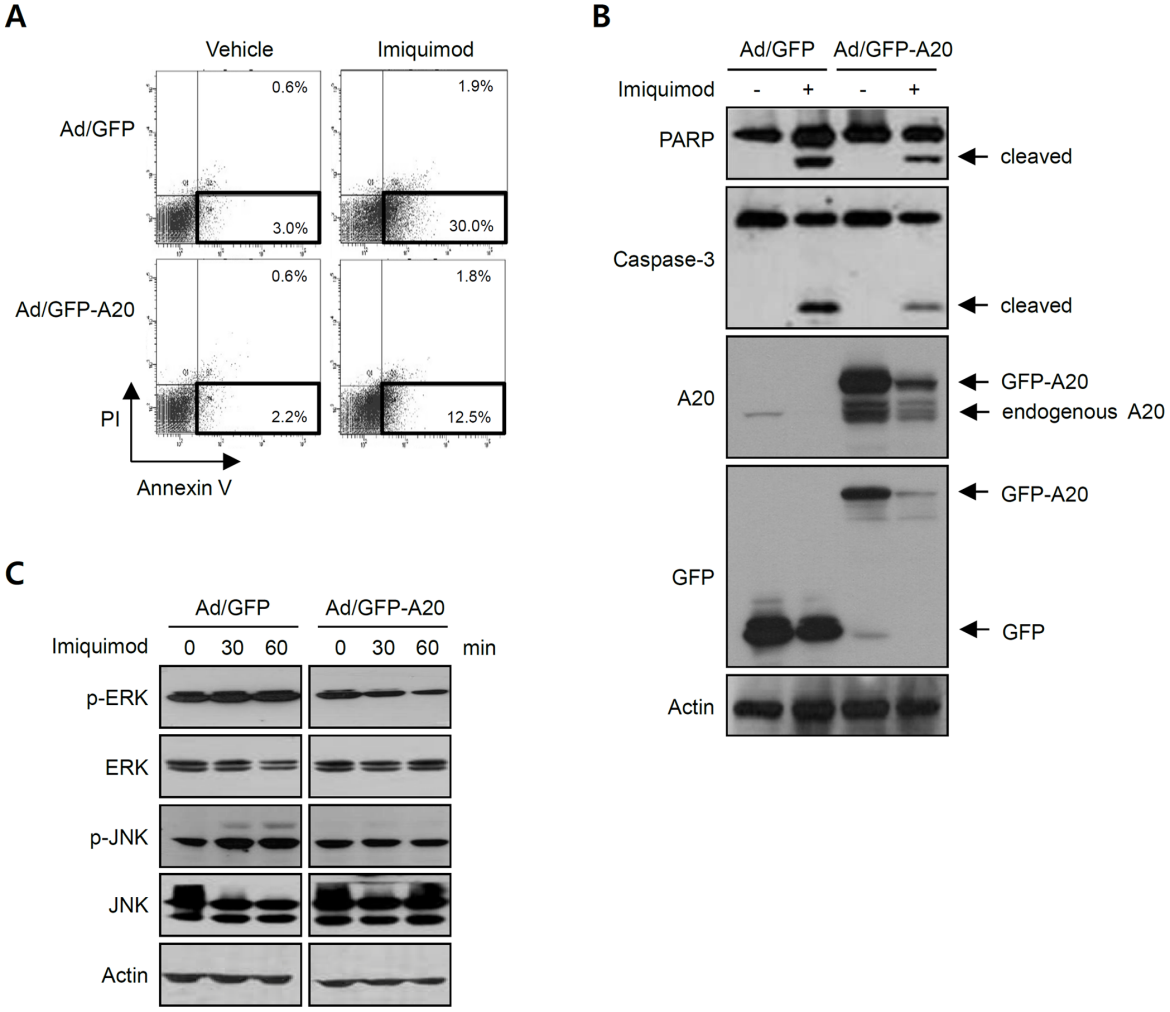
Effect of A20 overexpression on imiquimod-induced apoptosis of SCC12 cells. (A) Cells were transduced with 10 multiplicity of infection (MOI) of adenovirus expressing GFP-tagged A20 (Ad/GFP-A20) or control adenovirus (Ad/GFP) for 6 h. Cells were replenished with fresh medium, and incubated for a further 2 d. Then, cells were treated with imiquimod (150 µg/ml) for 16 h. Apoptosis was determined by flow cytometry. Annexin V high and propidium iodide (PI) dim cells (bold box) represent apoptotic cells. Imiquimod-induced apoptosis is markedly reduced in GFP-A20 overexpressed group (Ad/GFP-A20) compared to GFP overexpressed control group (Ad/GFP). (B) Cleavages of PARP and caspase-3 were detected by Western blot. In GFP-A20 overexpressed cells, imiquimod-driven PARP and caspase-3 cleavages are reduced compared to control group. (C) After adenoviral transduction, cells were treated with imiquimod (150 µg/ml) for the indicated time points, and phosphorylation of MAPKs was detected by Western blot. Imiquimod induces phosphorylation of JNK, which is inhibited by overexpression of GFP-A20.

To address a question whether A20 exerts antiapoptotic role, we exogenously overexpressed green fluorescent protein-tagged A20 (GFP-A20) using a recombinant adenovirus. Flow cytometry analysis showed that overexpression of A20 inhibited markedly the imiquimod-induced apoptosis (Figure 3A). Consistent with this data, imiquimod-induced cleavage of PARP and caspase-3 was significantly decreased by overexpression of A20 (Figure 3B). These results support the idea that down-regulation of A20 by imiquimod is linked to the apoptosis of SCC12 cells.

Recently, it has been demonstrated that A20 suppresses activation of c-Jun N-terminal kinase (JNK) by degrading apoptosis signal-regulated kinase 1 (ASK1), eventually leading to inhibition of apoptosis [17]. We wondered if similar signaling event occurs in imiquimod-induced apoptosis of SCC12 cells. Imiquimod treatment did not affect significantly the phosphorylation of extracellular signal-regulated kinase (ERK). On the contrary, imiquimod clearly activated JNK in terms of phosphorylation, while overexpression of A20 markedly inhibited activation of JNK by imiquimod (Figure 3C, Figure S5).

**Figure 4:**
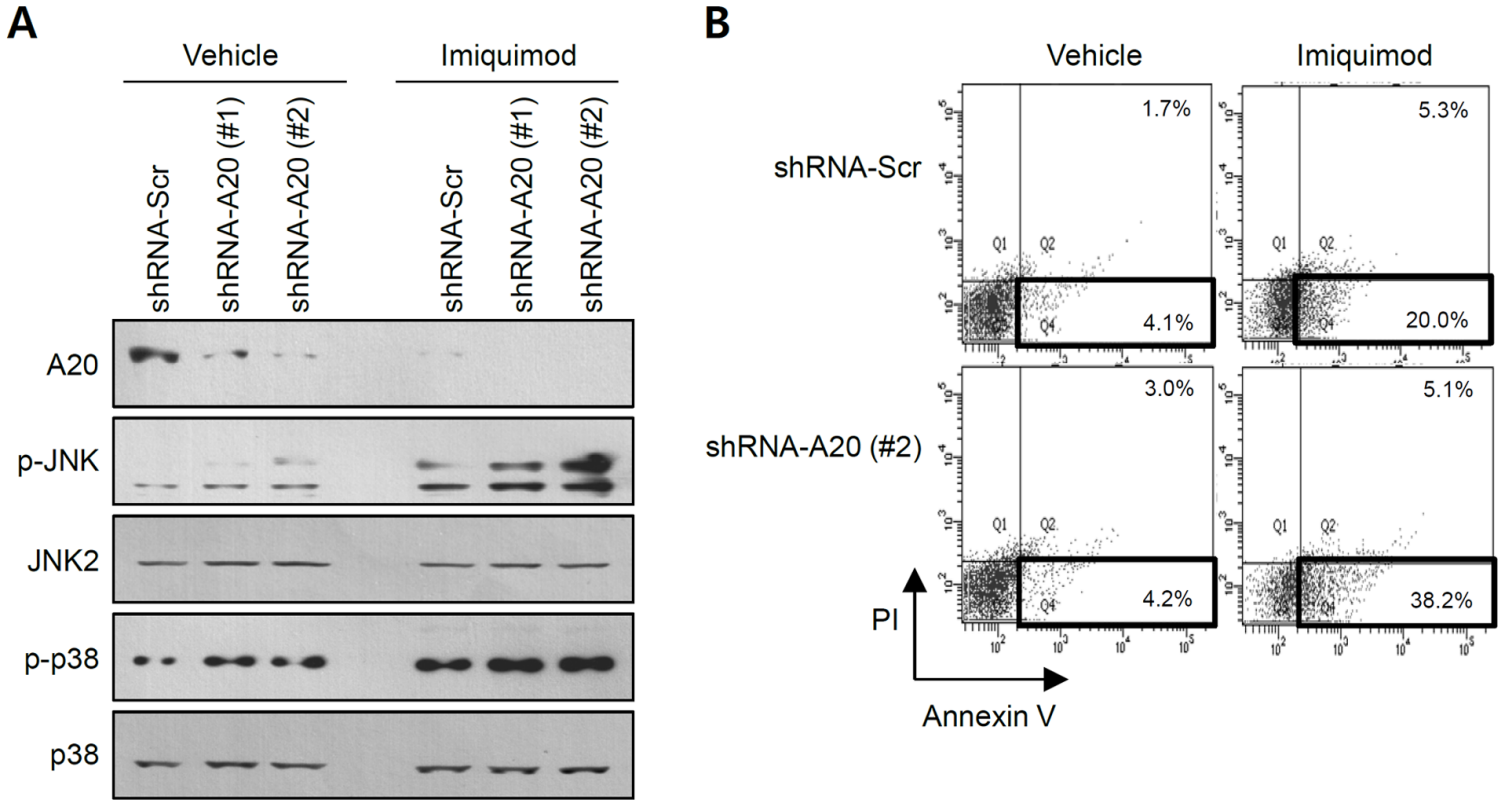
Effect of A20 knockdown on imiquimod-induced apoptosis of SCC12 cells. (A) The stable SCC12 cells expressing shRNA were established, and phosphorylation of MAPKs was detected by Western blot. In A20-knockdowned cells (shRNA-A20 (#1), shRNA-A20 (#2)), phosphorylation of JNK is increased compared to control cells (shRNA-Scr). (B) Cells were treated with imiquimod (150 µg/ml) for 16 h and apoptosis was determined by flow cytometry. Bold boxes represent apoptotic cells. Imiquimod-induced apoptosis is markedly potentiated in A20-knockdowned cells (shRNA-A20 (#2)) compared to control cells (shRNA-Scr).

To further address the role of A20 in imiquimod-induced apoptosis of SCC12 cells, we established the stable cell lines in which A20 expression was knockdowned by shRNA. Western blot showed that endogenous A20 expression was efficiently knockdowned by shRNA (Figure 4A). Consistent with previous data, knockdown of A20 led to increase of JNK phosphorylation. In addition, knockdown of A20 resulted in slight increase of phospho-p38 MAPK. Imiquimod treatment of A20 knockdowned-cells resulted in higher activation of JNK compared to control group (Figure 4A). As anticipated, knockdown of A20 potentiated the imiquimod-induced apoptosis (Figure 4B).

Collectively, these data strongly suggest that imiquimod-induced apoptosis is mediated through the suppression of A20 and activation of JNK. In line with this, pretreatment of SCC12 cells with JNK inhibitor (SP600125) significantly inhibited imiquimod-induced cell death (Figure S4), potentiating the idea that JNK activation mediates imiquimod-induced apoptosis in SCC12 cells.

### Imiquimod-induced Apoptosis of SCC12 Cells is Independent of TLR7

**Figure 5:**
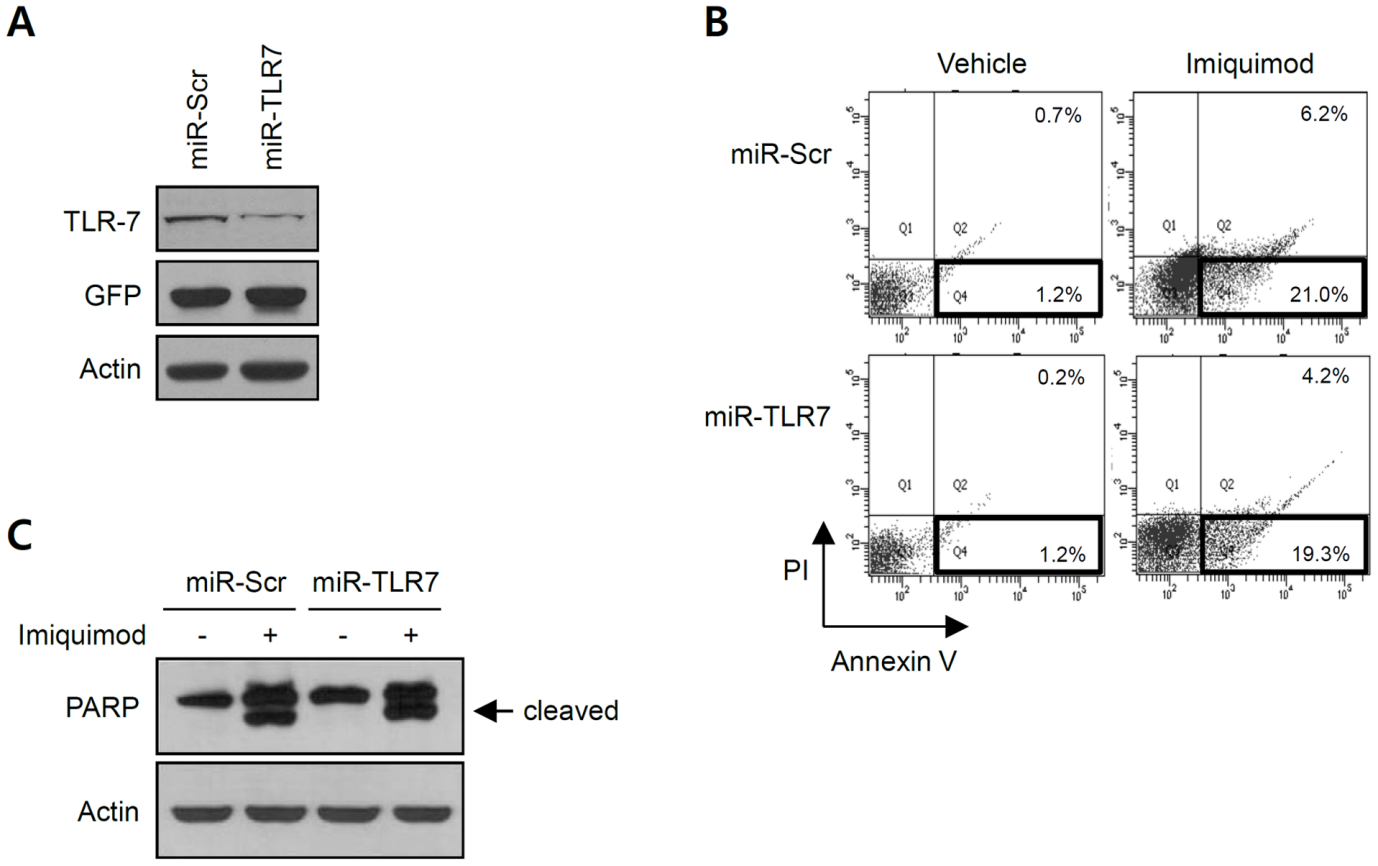
Effect of TLR7 knockdown on imiquimod-induced apoptosis of SCC12 cells. (A) Cells were transduced with 10 multiplicity of infection (MOI) of adenovirus expressing miR-TLR7 or control adenovirus (miR-Scr) for 6 h. Cells were replenished with fresh medium, and incubated for a further 2 d. Endogenous expression of TLR7 is markedly decreased by miR-TLR7. (B) After adenoviral transduction, cells were treated with imiquimod (150 µg/ml) for 16 h and apoptosis was determined by flow cytometry. Bold boxes represent apoptotic cells. There is no difference in apoptotic cell populations between TLR7-knockdowned group (miR-TLR7) and control group (miR-Scr). (C) Cleavages of PARP was detected by Western blot. TLR7 knockdown does not affect imiquimod-driven PARP cleavage.

Since imiquimod is a specific TLR7 ligand, we wondered if imiquimod-induced apoptosis of SCC12 cells was dependent of TLR7. To this end, we knockdowned TLR7 expression using a recombinant adenovirus expressing miR-TLR7 (Figure 5A). When TLR7 was knockdowned, imiquimod-induced apoptosis was not affected as compared with control group (Figure 5B). Consistent with this data, western blot showed that there was no difference in PARP cleavage between control and TLR7 knockdowned group (Figure 5C). These data suggest that imiquimod-induced apoptosis is not linked to the activation of TLR7 in SCC12 cells.

## Discussion

Imiquimod activates immune system thereby stimulating tumor destruction and/or preventing cancer growth. Besides its potential for inducing anti-tumor immunity, imiquimod can also affect cancer cells directly. In this study, we demonstrated that imiquimod induced apoptosis of SCC cells and antiapoptotic regulator A20 was involved in this process.

A20 is a negative regulator in NF-κB signaling pathway. A20 ubiquitinates receptor interacting protein 1 (RIP1), a critical signaling intermediate protein in tumor necrosis factor (TNF)-mediated NF-κB activation, resulting in proteasomal degradation of RIP1 and termination of NF-κB activation [23]. In this study, we showed that imiquimod treatment led to down-regulation of A20 in SCC12 cells. Thus it can be easily speculated that the modulation of NF-κB signaling is a putative underlying mechanism of imiquimod-induced apoptosis of SCC12 cells. NF-κB is normally present in almost all animal cells as an inactive form, however it is activated by various stimuli such as UV radiation, free radicals and microbial antigens [24]–[26]. It has been reported that constitutive activation of NF-κB occurs in colorectal, pancreatic and liver cancers, suggesting NF-κB is a causative player in progression of diverse malignant neoplasms [27]–[29]. On the contrary, NF-κB shows opposite effect in skin epithelial cells. For example, SCC occurs spontaneously when NF-κB signaling is selectively inhibited by overexpression of IκB-α super-repressor form [5]. In other example, overexpression of active p50 and p65 NF-κB subunits in transgenic epithelium produces hypoplasia and growth inhibition, while functional blockade of NF-κB by expressing dominant-negative NF-κB inhibitory proteins in transgenic murine and human epidermis produces hyperplastic epithelium in vivo [30]. Thus, it is tempting to explain that activation of NF-κB signaling negatively affects the proliferation of skin keratinocytes, eventually leading to apoptosis. Since A20 is a well-established feedback inhibitor for NF-κB activation, it can be suggest that imiquimod-induced down-regulation of A20 contributes to NF-κB activation, thereby leading to cell growth inhibition and apoptosis of SCC12 cells.

In this study, imiquimod induced activation of JNK, which was effectively inhibited by overexpression of A20. Conversely, knockdown of A20 resulted in JNK activation. Thus it can be suggested that JNK activation is a consequence of imiquimod-driven A20 down-regulation. Interestingly, it has been demonstrated that A20 degrades ASK1, an upstream kinase for JNK activation [17]. Thus, there is a possibility that ASK1 can be a link between imiquimod and JNK activation. In our preliminary experiment, imiquimod treatment led to significant increase of phsopho-ASK1, and overexpression of A20 markedly inhibited the imiquimod-induced phosphorylation of ASK1 (Figure S5), supporting the idea that ASK1 is involved in imiquimod-induced JNK activation. Since it has been shown that persistent JNK activation contributes to TNF-induced apoptosis [31], it is likely that A20-regulation on JNK activity is a key process in imiquimod-induced apoptosis of SCC12 cells.

It is thought that imiquimod-induced apoptosis of SCC12 cells is independent of TLR7, a well-established receptor for imiquimod. Interestingly, previous report indicates that imiquimod induces activation of NF-κB and the downstream production of proinflammatory cytokines in the absence of TLR7. TLR-independent effects of imiquimod have been suggested to stem from its interference with adenosine receptor signaling mediated by adenylyl cyclase. In addition, imiquimod exerts direct or indirect adenosine receptor-independent inhibition of adenylyl cyclase activity [32]. Because that knockdown of TLR7 did not block imiquimod-driven apoptosis in our study, it is assumption that other transmembrane receptors, such as adenosine receptor, are involved in imiquimod-induced apoptosis of SCC12 cells. Elucidation of TLR-independent mechanism will be an interesting further study.

In summary, we demonstrate that imiquimod induces apoptosis of SCC cells, and that A20 is a critical player in this process. Our results may contribute to a better understanding of action mechanism of imiquimod on cutaneous cancers, and may help to develop new target for SCC.

## Supporting Information

Figure S1 Expression of NF-κB target genes in squamous cell carcinoma (SCC) cells. Cellular extracts were prepared and expression of NF-κB target genes was validated using Western blot. As compared to normal human epidermal keratinocytes (NHEK), SCC12 cells show higher expression of several NF-κB target genes.
(PDF)

Figure S2 Expression of A20 in squamous cell carcinoma (SCC) tissues. For simultaneous detection of A20 expression, tissue array analysis was performed. The moderate to high expression of A20 (P 1∼P 11) is observed in about 78% (11/14) patient samples.
(PDF)

Figure S3 Effect of A20 overexpression on imiquimod-induced apoptosis of SCC12 cells. Cells were transduced with adenovirus expressing GFP-tagged A20 (Ad/GFP-A20) or control adenovirus (Ad/GFP), then treated with imiquimod. Caspase activation was determined by Western blot. Cleavage of caspase-9, but not caspase-8, was detected, suggesting that imiquimod induces activation of intrinsic apoptotic pathway.
(PDF)

Figure S4 SCC12 cells were pretreated with JNK inhibitor SP600125 (20 µM), then treated with imiquimod (150 µg/ml). After 24 h incubation, cell viability was determined by MTT assay. Data are expressed as percentage of control. The mean values ± SD are averages of triplicate measurements. (*P<0.01).
(PDF)

Figure S5 SCC12 cells were transduced with adenovirus expressing GFP-tagged A20 (Ad/GFP-A20) or control adenovirus (Ad/GFP), then treated with imiquimod (150 µg/ml) for the indicated time points. Phosphorylation of ASK1 and MAPKs was detected by Western blot. Imiquimod induces phosphorylation of ASK1 and JNK, which is inhibited by overexpression of GFP-A20.
(PDF)

## References

[1] Alam M, Ratner D (2001) Cutaneous squamous-cell carcinoma. N Engl J Med 344: 975–983. 11274625 10.1056/NEJM200103293441306

[2] Brash DE, Rudolph JA, Simon JA, Lin A, McKenna GJ, et al (1991) A role for sunlight in skin cancer: UV-induced p53 mutations in squamous cell carcinoma. Proc Natl Acad Sci USA 88: 10124–10128. 1946433 10.1073/pnas.88.22.10124 PMC52880

[3] Kolev V, Mandinova A, Guinea-Viniegra J, Hu B, Lefort K, et al (2008) EGFR signalling as a negative regulator of Notch1 gene transcription and function in proliferating keratinocytes and cancer. Nat Cell Biol 10: 902–911. 18604200 10.1038/ncb1750 PMC2747621

[4] Zhao L, Li W, Marshall C, Griffin T, Hanson M, et al (2009) Srcasm inhibits Fyn-induced cutaneous carcinogenesis with modulation of Notch1 and p53. Cancer Res 69: 9439–9447. 19934324 10.1158/0008-5472.CAN-09-2976 PMC2794931

[5] van Hogerlinden M, Rozell BL, Ahrlund-Richter L, Toftgard R (1999) Squamous cell carcinomas and increased apoptosis in skin with inhibited Rel/nuclear factor-κB signaling. Cancer Res 59: 3299–3303. 10416581

[6] Dajee M, Lazarov M, Zhang JY, Cai T, Green CL, et al (2003) NF-κB blockade and oncogenic Ras trigger invasive human epidermal neoplasia. Nature 421: 639–643. 12571598 10.1038/nature01283

[7] Stanley MA (2002) Imiquimod and the imidazoquinolones: mechanism of action and therapeutic potential. Clin Exp Dermatol 27: 571–577. 12464152 10.1046/j.1365-2230.2002.01151.x

[8] Schiller M, Metze D, Luger TA, Grabbe S, Gunzer M (2006) Immune response modifiers-mode of action. Exp Dermatol 15: 331–341. 16630072 10.1111/j.0906-6705.2006.00414.x

[9] Bong AB, Bonnekoh B, Franke I, Schön M, Ulrich J, et al (2002) Imiquimod, a topical immune response modifier, in the treatment of cutaneous metastases of malignant melanoma. Dermatology 205: 135–138. 12218228 10.1159/000063904

[10] Geisse JK, Rich P, Pandya A, Gross K, Andres K, et al (2002) Imiquimod 5% cream for the treatment of superficial basal cell carcinoma: a double-blind, randomized, vehicle-controlled study. J Am Acad Dermatol 47: 390–398. 12196749 10.1067/mjd.2002.126215

[11] Stary G, Bangert C, Tauber M, Strohal R, Kopp T, et al (2007) Tumoricidal activity of TLR7/8-activated inflammatory dendritic cells. J Exp Med 204: 1441–1451. 17535975 10.1084/jem.20070021 PMC2118597

[12] Huang SJ, Hijnen D, Murphy GF, Kupper TS, Calarese AW, et al (2009) Imiquimod enhances IFN-gamma production and effector function of T cells infiltrating human squamous cell carcinomas of the skin. J Invest Dermatol 129: 2676–2685. 19516264 10.1038/jid.2009.151 PMC2841955

[13] Yokogawa M, Takaishi M, Nakajima K, Kamijima R, Digiovanni J, et al (2013) Imiquimod attenuates the growth of UVB-Induced SCC in mice through Th1/Th17 cells. Mol Carcinog 52: 760–769. 22431065 10.1002/mc.21901 PMC3397281

[14] Schön MP, Schön M, Klotz KN (2006) The small antitumoral immune response modifier imiquimod interacts with adenosine receptor signaling in a TLR7- and TLR8-independent fashion. J Invest Dermatol 126: 1338–1347. 16575388 10.1038/sj.jid.5700286

[15] Lee JS, Kim DH, Choi DK, Kim CD, Ahn GB, et al (2013) Comparison of Gene Expression Profiles between Keratinocytes, Melanocytes and Fibroblasts. Ann Dermatol 25: 36–45. 23467683 10.5021/ad.2013.25.1.36 PMC3582926

[16] Shi G, Sohn KC, Li Z, Choi DK, Park YM, et al (2013) Expression and functional role of Sox9 in human epidermal keratinocytes. PLoS One 8: e54355. 23349860 10.1371/journal.pone.0054355 PMC3548846

[17] Won M, Park KA, Byun HS, Sohn KC, Kim YR, et al (2010) Novel anti-apoptotic mechanism of A20 through targeting ASK1 to suppress TNF-induced JNK activation. Cell Death Differ 17: 1830–1841. 20448643 10.1038/cdd.2010.47 PMC7386326

[18] Li ZJ, Sohn KC, Choi DK, Shi G, Hong D, et al (2013) Roles of TLR7 in activation of NF-κB signaling of keratinocytes by imiquimod. PLoS One 8: e77159. 24146965 10.1371/journal.pone.0077159 PMC3795621

[19] Balkwill F, Coussens LM (2004) Cancer: an inflammatory link. Nature 431: 405–406. 15385993 10.1038/431405a

[20] Kobielak A, Fuchs E (2006) Links between α-catenin, NF-κB, and squamous cell carcinoma in skin. Proc Natl Acad Sci USA 103: 2322–2327. 16452166 10.1073/pnas.0510422103 PMC1413714

[21] Lee EG, Boone DL, Chai S, Libby SL, Chien M, et al (2000) Failure to regulate TNF-induced NF-κB and cell death responses in A20-deficient mice. Science 289: 2350–2354. 11009421 10.1126/science.289.5488.2350 PMC3582399

[22] Renner F, Schmitz ML (2009) Autoregulatory feedback loops terminating the NF-κB response. Trends Biochem Sci 34: 128–135. 19233657 10.1016/j.tibs.2008.12.003

[23] Shembade N, Harhaj EW (2012) Regulation of NF-κB signaling by the A20 deubiquitinase. Cell Mol Immunol 9: 123–130. 22343828 10.1038/cmi.2011.59 PMC3532050

[24] Gilmore TD (2006) Introduction to NF-κB: players, pathways, perspectives. Oncogene 25: 6680–6684. 17072321 10.1038/sj.onc.1209954

[25] Baldwin AS Jr (1996) The NF-κB and IκB proteins: new discoveries and insights. Annu Rev Immunol 14: 649–683. 8717528 10.1146/annurev.immunol.14.1.649

[26] Garg A, Aggarwal BB (2002) Nuclear transcription factor-κB as a target for cancer drug development. Leukemia 16: 1053–1068. 12040437 10.1038/sj.leu.2402482

[27] Yu HG, Yu LL, Yang Y, Luo HS, Yu JP, et al (2003) Increased expression of RelA/nuclear factor-κB protein correlates with colorectal tumorigenesis. Oncology 65: 37–45. 10.1159/000071203 12837981

[28] Liptay S, Weber CK, Ludwig L, Wagner M, Adler G, et al (2003) Mitogenic and antiapoptotic role of constitutive NF-κB/Rel activity in pancreatic cancer. Int J Cancer 105: 735–746. 12767057 10.1002/ijc.11081

[29] Li W, Tan D, Zenali MJ, Brown RE (2009) Constitutive activation of nuclear factor-kappa B (NF-κB) signaling pathway in fibrolamellar hepatocellular carcinoma. Int J Clin Exp Pathol 3: 238–243. 20224721 PMC2836501

[30] Seitz CS, Lin Q, Deng H, Khavari PA (1998) Alterations in NF-κB function in transgenic epithelial tissue demonstrate a growth inhibitory role for NF-κB. Proc Natl Acad Sci USA 95: 2307–2312. 9482881 10.1073/pnas.95.5.2307 PMC19329

[31] Sakon S, Xue X, Takekawa M, Sasazuki T, Okazaki T, et al (2003) NF-κB inhibits TNF-induced accumulation of ROS that mediate prolonged MAPK activation and necrotic cell death. EMBO J 22: 3898–3909. 12881424 10.1093/emboj/cdg379 PMC169052

[32] Schön MP, Schön M, Klotz KN (2006) The small antitumoral immune response modifier imiquimod interacts with adenosine receptor signaling in a TLR7- and TLR8-independent fashion. J Invest Dermatol 126: 1338–1347. 16575388 10.1038/sj.jid.5700286

