# Supplementary figures and images for: Imiquimod Induces Apoptosis of Squamous Cell Carcinoma (SCC) Cells via Regulation of A20
Source: PLoS One. 2014 Apr 17;9(4):e95337. doi: 10.1371/journal.pone.0095337 (PMC3990708; doi:10.1371/journal.pone.0095337)

**Figure S1**

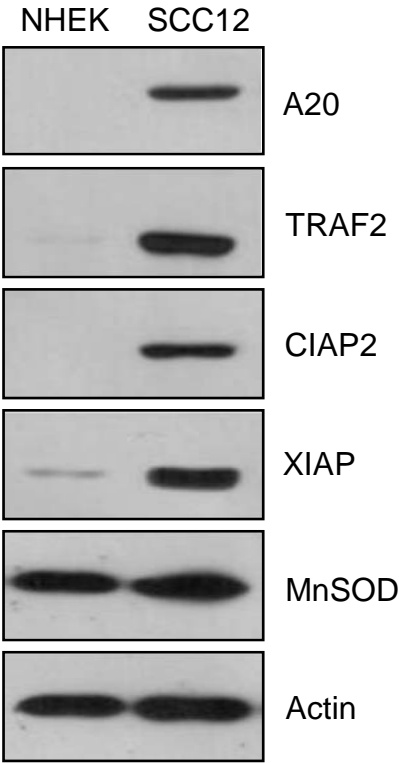

Supplement: Figure S1 — Expression of NF-κB target genes in squamous cell carcinoma (SCC) cells. Cellular extracts were prepared and expression of NF-κB target genes was validated using Western blot. As compared to normal human epidermal keratinocytes (NHEK), SCC12 cells show higher expression of several NF-κB target genes. (PDF) [file pone.0095337.s001.pdf]

**Figure S2**

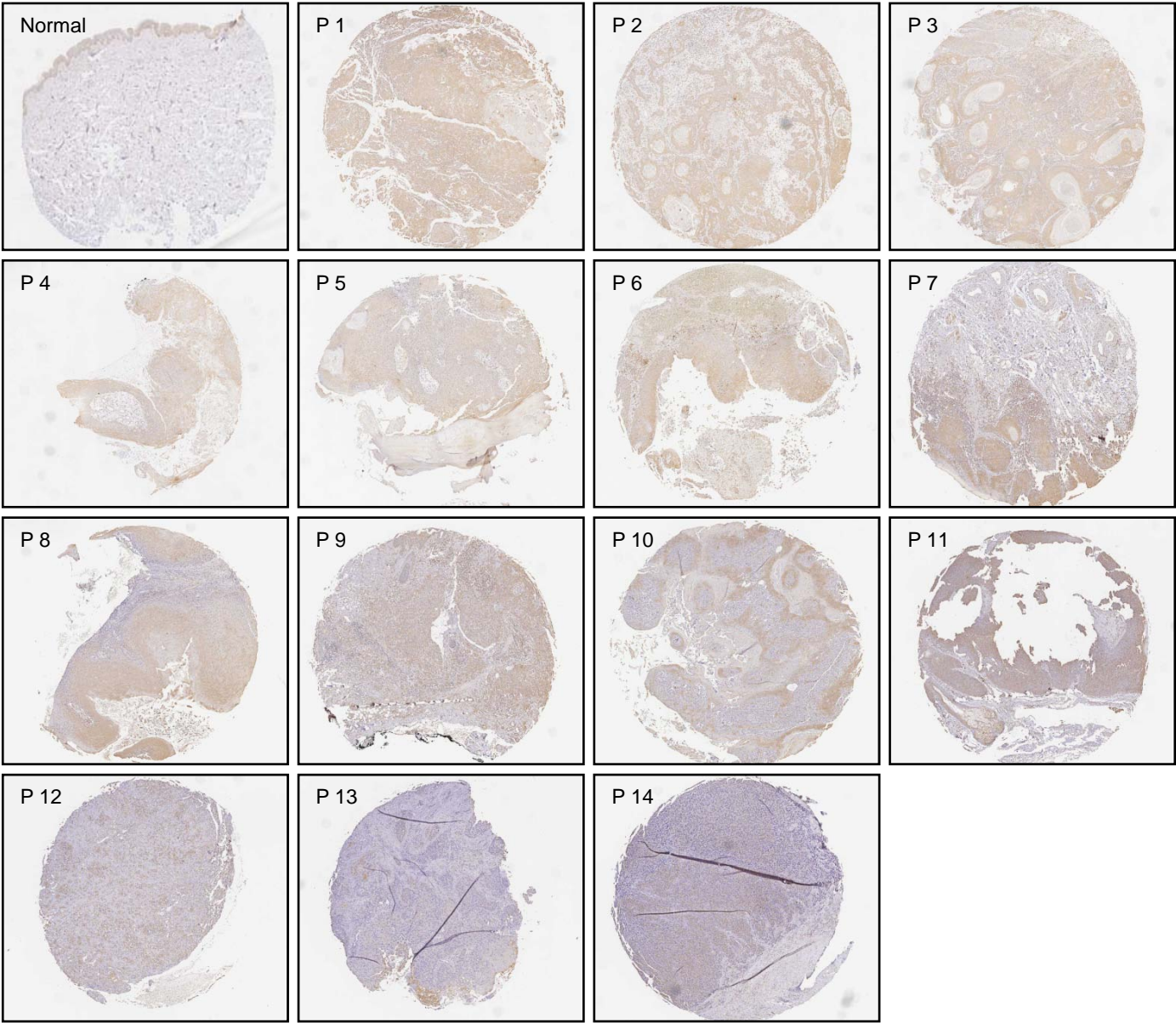

Supplement: Figure S2 — Expression of A20 in squamous cell carcinoma (SCC) tissues. For simultaneous detection of A20 expression, tissue array analysis was performed. The moderate to high expression of A20 (P 1∼P 11) is observed in about 78% (11/14) patient samples. (PDF) [file pone.0095337.s002.pdf]

Figure S3

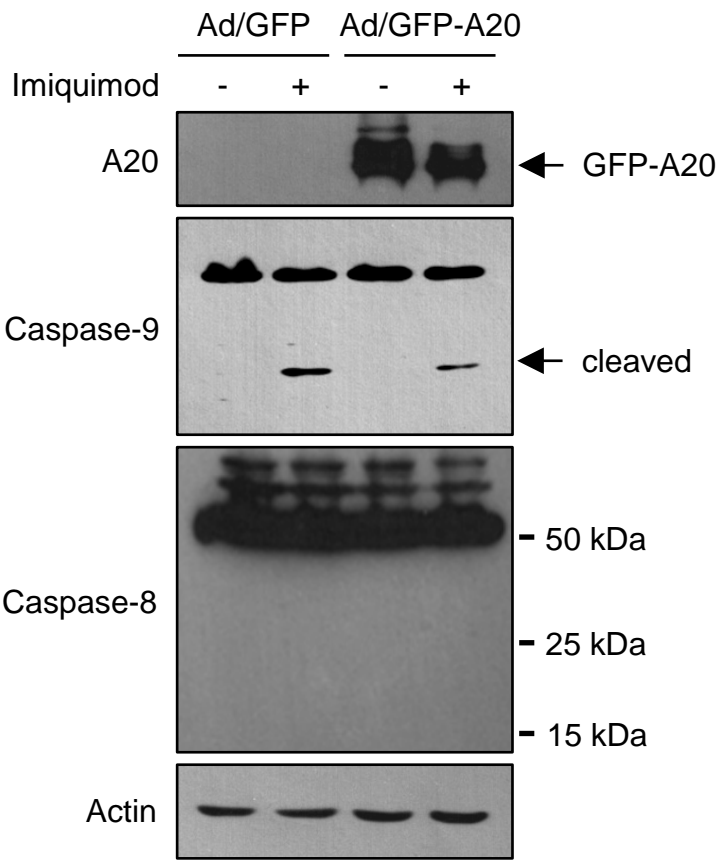

Supplement: Figure S3 — Effect of A20 overexpression on imiquimod-induced apoptosis of SCC12 cells. Cells were transduced with adenovirus expressing GFP-tagged A20 (Ad/GFP-A20) or control adenovirus (Ad/GFP), then treated with imiquimod. Caspase activation was determined by Western blot. Cleavage of caspase-9, but not caspase-8, was detected, suggesting that imiquimod induces activation of intrinsic apoptotic pathway. (PDF) [file pone.0095337.s003.pdf]

**Figure S4**

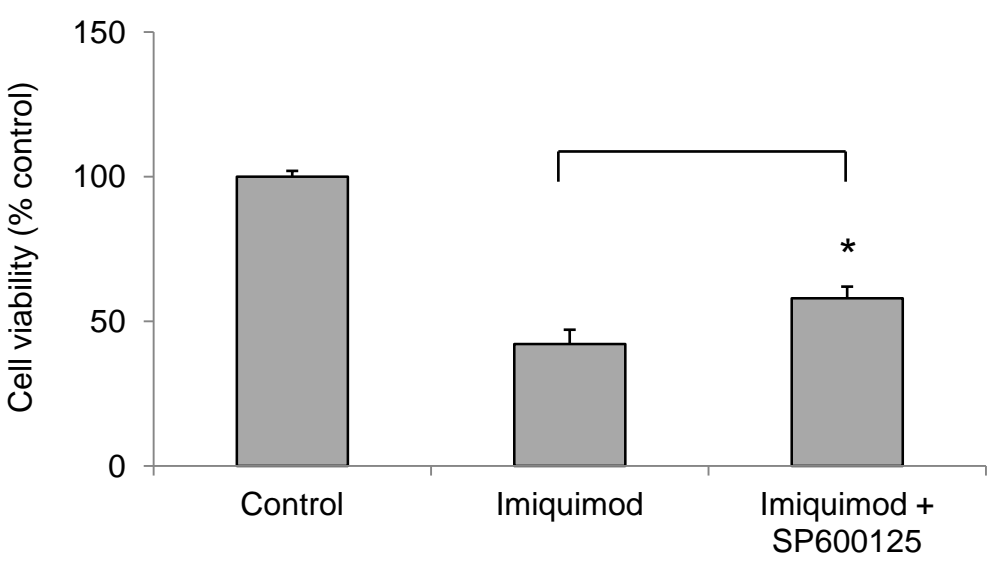

Supplement: Figure S4 — SCC12 cells were pretreated with JNK inhibitor SP600125 (20 µM), then treated with imiquimod (150 µg/ml). After 24 h incubation, cell viability was determined by MTT assay. Data are expressed as percentage of control. The mean values ± SD are averages of triplicate measurements. (*P<0.01). (PDF) [file pone.0095337.s004.pdf]

**Figure S5**

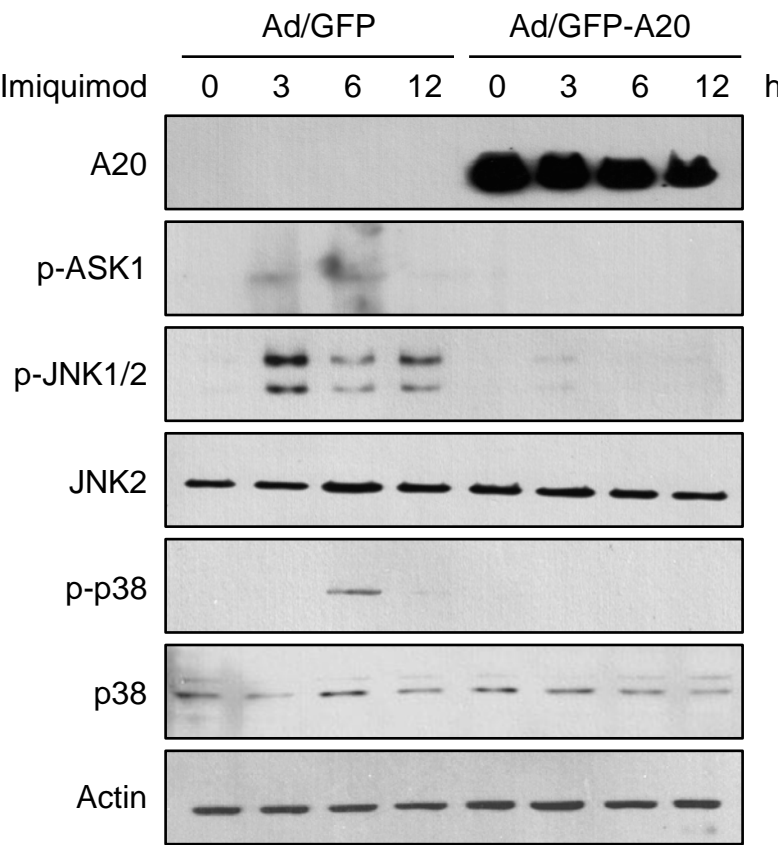

Supplement: Figure S5 — SCC12 cells were transduced with adenovirus expressing GFP-tagged A20 (Ad/GFP-A20) or control adenovirus (Ad/GFP), then treated with imiquimod (150 µg/ml) for the indicated time points. Phosphorylation of ASK1 and MAPKs was detected by Western blot. Imiquimod induces phosphorylation of ASK1 and JNK, which is inhibited by overexpression of GFP-A20. (PDF) [file pone.0095337.s005.pdf]
